# Feasibility of intratumoral ^165^Holmium siloxane delivery to induced U87 glioblastoma in a large animal model, the Yucatan minipig

**DOI:** 10.1371/journal.pone.0234772

**Published:** 2020-06-18

**Authors:** Mehrdad Khoshnevis, Claude Carozzo, Richard Brown, Manuel Bardiès, Catherine Bonnefont-Rebeix, Sara Belluco, Christophe Nennig, Lionel Marcon, Olivier Tillement, Hélène Gehan, Cédric Louis, Ilyes Zahi, Thierry Buronfosse, Thierry Roger, Frédérique Ponce

**Affiliations:** 1 ICE (Interactions Cellules Environnement), UPSP 2016.A104, VetAgro Sup, University of Lyon1, Marcy l’Etoile, France; 2 Inserm, UMR1037 CRCT, Toulouse, France; 3 EVEON, Montbonnot Saint Martin, France; 4 Institut Lumière Matière, UMR CNRS 5306, UCBL, Campus LyonTech—La Doua, Villeurbanne, France; 5 Nano-H SAS, Saint-Quentin Fallavier, France; 6 Advanced Accelerator Applications, Saint-Genis Pouilly, France; 7 Department of Endocrinology, VetAgro Sup, University of Lyon1, Marcy l’Etoile, France; 8 Clinical Oncology Unit, VetAgro Sup, University of Lyon1, Marcy l’Etoile, France; George Washington University, UNITED STATES

## Abstract

Glioblastoma is the most aggressive primary brain tumor leading to death in most of patients. It comprises almost 50–55% of all gliomas with an incidence rate of 2–3 per 100,000. Despite its rarity, overall mortality of glioblastoma is comparable to the most frequent tumors. The current standard treatment combines surgical resection, radiotherapy and chemotherapy with temozolomide. In spite of this aggressive multimodality protocol, prognosis of glioblastoma is poor and the median survival remains about 12–14.5 months. In this regard, new therapeutic approaches should be developed to improve the life quality and survival time of the patient after the initial diagnosis. Before switching to clinical trials in humans, all innovative therapeutic methods must be studied first on a relevant animal model in preclinical settings. In this regard, we validated the feasibility of intratumoral delivery of a holmium (Ho) microparticle suspension to an induced U87 glioblastoma model. Among the different radioactive beta emitters, ^166^Ho emits high-energy β(-) radiation and low-energy γ radiation. β(-) radiation is an effective means for tumor destruction and γ rays are well suited for imaging (SPECT) and consequent dosimetry. In addition, the paramagnetic Ho nucleus is a good asset to perform MRI imaging. In this study, five minipigs, implanted with our glioblastoma model were used to test the injectability of ^165^Ho (stable) using a bespoke injector and needle. The suspension was produced in the form of Ho microparticles and injected inside the tumor by a technique known as microbrachytherapy using a stereotactic system. At the end of this trial, it was found that the ^165^Ho suspension can be injected successfully inside the tumor with absence or minimal traces of Ho reflux after the injections. This injection technique and the use of the ^165^Ho suspension needs to be further assessed with radioactive ^166^Ho in future studies.

## Introduction

Glioblastoma (GB) is an inherently aggressive tumor and by far the most frequent and lethal malignant primary brain tumor in adults [1–4]. It constitutes almost 25% of all primary central nervous system (CNS) tumors and 50–55% of the malignant gliomas [5,6]. GB has a high incidence among all malignant brain tumors, with an estimation of 10,000–12,000 new cases per year [5,7,8]. GB incidence has increased during the past 20 years, mainly due to the improvement of imaging diagnosis, such as CT-scans and MRI [9,10]. GB develops into a highly invasive metastasis, mainly located in the CNS [11–14].

The standard treatments include surgery, radiotherapy (RT) and chemotherapy [5,14–16]. The gold-standard treatment protocol is a maximal surgical resection of the tumor, plus RT with concurrent and adjuvant medical chemotherapy, such as Temozolomide (TMZ) [5]. New treatments, such as immunotherapy, have emerged but results for these new modalities are not yet conclusive [14–17]. Unfortunately, GB is associated with an extremely poor prognosis [7,18,19] and patients are often confronted with a recurrence of the disease after a few months [2,15,20,21].

Today, in addition to surgery, RT is also the foundation of GB treatment [22–25]. During the 1970s, the dose-effect relationship in RT of GB was shown by Walker and colleagues [24,26]. They showed that radiation dose of 60 Gy was associated with increased survival-times in comparison with other doses and without significantly increased toxicity [27,28]. Dose escalation over 60 Gy resulted in increased toxicity without survival benefit [14,29]. Two main types of RT that are relevant to this research are 1) External beam radiation therapy (EBRT), such as 3DCRT, IMRT, VMAT and SRS, 2) brachytherapy (BT).

EBRT is a precise modality that directs a beam of radiation from outside the patient. This means that the radiation passes through healthy tissue before being delivered to the tumor and thus creates further non-desired destructive damages to them.

BT is defined as a short-distance treatment for malignant tumors. It consists of placing sealed radioactive sources inside or next to the tumor to deliver ionizing radiation into the area requiring treatment [30]. BT can use temporarily or permanently placed radioactive sources. Treatment can be carried out with the placement of radioactive seeds or even with the implantation of a balloon filled with radioactive elements. The balloon device gives some advantages, such as ease of use, although the treatment field is restricted to spherical contours. This constraint is solved by implanting the radioactive seed, but the dosimetry related to seed implants is usually more complex [31–33].

The radio-resistance of GB makes its treatment more difficult. Intratumoral hypoxia and the presence of microglia have an important role in the resistance to radiotherapy [34–38].

Nonetheless, adjuvant RT still has a key role in GB treatment with a significant survival benefit when compared with different chemotherapy protocols without RT [39–41]. Unfortunately, RT only delays but does not normally prevent recurrence, which usually occurs within 2 cm of the initial tumor site [5,39,42,43].

Further developments to radiotherapy-based treatments are required to improve the median survival of GB patients [24]. The desire to increase the irradiation of the tumor whilst sparing organs at risk (OARs) has led to the development of an extension of BT, known as microbrachytherapy. Instead of placing radioactive seeds as with BT, this technique uses microspheres or microparticles in suspension containing a highly concentrated radioactive agent. Since microbrachytherapy enables a higher specific activity into the tumor and better spares surrounding healthy tissue, it could be beneficial for patients, increasing overall survival and decreasing toxicity.

Experimental tests with this method can determine its efficacy with regards to the treatment of GB. However, before testing the efficacy of this new modality, a method of delivery and injection of radioactive microspheres must first be developed. Such methodology is proposed and discussed in this article.

Holmium (Ho) was selected because it has some advantages over other elements; 1) Ho is a highly paramagnetic isotope, suitable for MRI [44,45], 2) activated holmium (^166^Ho) is easily obtained following neutron-activation thanks to an adequate neutron capture nuclear reaction known as “cross section” parameter, 3) ^166^Ho has a short half-life 26.8 hours and decays rapidly to stable products, 4) ^166^Ho deposes 90% of the energy within the range of 2.1 mm and remaining 10% within the range of 2.1–8.6 mm, suggesting that it can intensively irradiate inside the tumor only, whilst preserving the surrounding normal tissue, 5) ^166^Ho emits high-energy (1840 MeV) β(-) radiation and low-energy (81 keV) γ radiation [46–49]. β(-) is an effective means for tumor cell destruction and γ can be used for post-treatment dosimetry and/or patient follow-up thanks to SPECT imaging.

In a previous study [50], injectability and preliminary acute toxicity of the non-radioactive and stable isotope of holmium (^165^Ho) suspension were assessed after stereotactic injection in the brain of a healthy minipig. The use of ^165^Ho allows testing the delivery methodology before adding the radioactive isotope, ^166^Ho. It was demonstrated that ^165^Ho suspension was suitable for intracerebral delivery and could potentially enable the delivery of high ^166^Ho concentration to the site of action *via* stereotactic injection.

Because the density of GB tumors is different from healthy brain tissue, a second step was needed to evaluate and validate the administration of ^165^Ho suspension inside GB tumors, before using the radioactive, ^166^Ho. The use of ^165^Ho as a stable isotope of Ho enables to overcome difficulties regarding the handling of radioactivity during this test, such as the necessity of radioprotection and the possibility of radioactive contamination. The aim of our study was to validate the intratumoral injectability of suspended microparticles containing ^165^Ho on a Yucatan minipig GB model [7].

## Materials and methods

### Ethics statement

The experiments were conducted in agreement with the French Ministry of Scientific Research (2015052012034148 v1) and approved by the VetAgro Sup Ethical Committee (1522 V2). All aspects of care and use of the animals, including surgical procedures and pain assessment were carried out and monitored in compliance with French regulations (transposition of Directive 2010/63/EU) and the local Animal Welfare Body.

### Animals

Yucatan minipig was used as a GB model for this study. Five minipigs, between 3–4 months old, both male and female, each weighing about 10 kg, were obtained from INRA Saint-Gilles, France and were moved to the preclinical platform of VetAgro Sup named Claude Bourgelat Institute in Marcy l’Etoile, France. All minipigs were identified by ear tag with an individual number. An acclimatization period of 7–10 days was allowed to reduce the transportation stress and to check any congenital or infectious diseases before administration of the immunosuppressive treatment. Environment-enrichment devices, such as plastic balls and toys were also provided. The minipigs were monitored 3 times each day. Daily examinations of the animals were performed by a veterinarian. Any focal infection was a criterion for exclusion from the study. Once per week, blood samples were collected from the pigs to analysis their blood parameters. The maintenance conditions of the minipigs were as follows: temperature = 19°C (± 2°C), humidity >35% and ventilation 10 times/hour. The feed was provided by the breeder, with an intake of 350–400 g/day/animal (adjusted according to growth). All the animals had unlimited access to water with nipple drinkers installed inside the cage. Their efficiency was tested every day.

### U87 cells preparation

U87 cells were chosen for development of GB in Yucatan minipig because they were used in previous studies on Landrace pigs and Yucatan minipigs [7,51]. Cells were obtained from the American Type Culture Collection (U-87 MG, ATCC® HTB-14™, LGC Standards, Molsheim, France). Regarding the ATCC product sheet, mycoplasma contamination was eliminated in September 1975 [52]. For inoculation of U87 cells in each subject, U87 cells were plated in 2 T175 tissue culture flasks with a density of 0.8–1.2 × 10^6^ cells per flask. Cells were grown in Minimum Essential Media (MEM) medium supplemented with 10% fetal calf serum, 100 U/ml penicillin, 100 μg/ml streptomycin, 2 mM l-Glutamine and 0.25% g/mL amphotericin B. Cells were maintained at 37°C in a humidified CO2 (5%) atmosphere, and the medium was changed every 3 days. A week later, cells from these flasks were trypsinized with 0.25% trypsin-EDTA and then 35–50 × 10^6^ cells per flask were harvested. Cells were washed twice in PBS before being pelleted 4 min at 3000 g in a 2 ml Eppendorf vial just before the inoculations. The final cell concentration in each Eppendorf vial was about 1.7 ×10^6^ cells/10μl. For each subject, 40 μl and 20 μl of the cells pellet were drawn up into the syringe and injected into the right and left hemisphere, respectively.

### Holmium

^165^Ho siloxane particles with a high ^165^Ho content suitable for microbrachytherapy of brain cancer were used (Nano-H SAS, Saint-Quentin Fallavier, France). For the preparation of ^165^Ho siloxane particles, nanostructured Ho_2_O_3_ precursor (0.397 mol) was slurried and refluxed in 1.5 L ethanol together with acetic acid (0.262 mol) and Si-EDTA (0.05 mol). It was allowed to cool down to ambient temperature and then transferred into 200 mL plastic centrifuge bottles. Particles were washed in ethanol twice by centrifugation cycles of 10 minutes at 4100 rpm and then resuspended in 160 mL mQ water. Particle size was homogenized by stirring the suspension for 4 days at 30°C. The objective was to create about 400 nm particles with high ^165^Ho content. In this study, the particle size was 470 nm, as measured by Dynamic Light Scattering (DLS). The concentration of dry matter in the suspension was 550 ± 50 g/L and the suspension had an Ho content of 28%. This value will potentially enable the delivery of a high absorbed dose to tumor cells in case of administration of activated suspension. Density of the final ^165^Ho siloxane suspension was 1.38 g/ml, measured in triplicate at 25°C by weighing 1 ml of suspension.

### Stereotactic frame

A large animal stereotactic frame (RWD 68901) was selected for intracerebral inoculation of tumoral cells and intratumoral injections of Ho. The frame and its manipulator can be used for neuroscience experiments on most large laboratory animals. This system enables the neurosurgeon to perform precise injections directly into targeted tissue. The location of the injection is obtained with a pre-operative CT-Scan.

### Preclinical injection system

**Fig 1:**
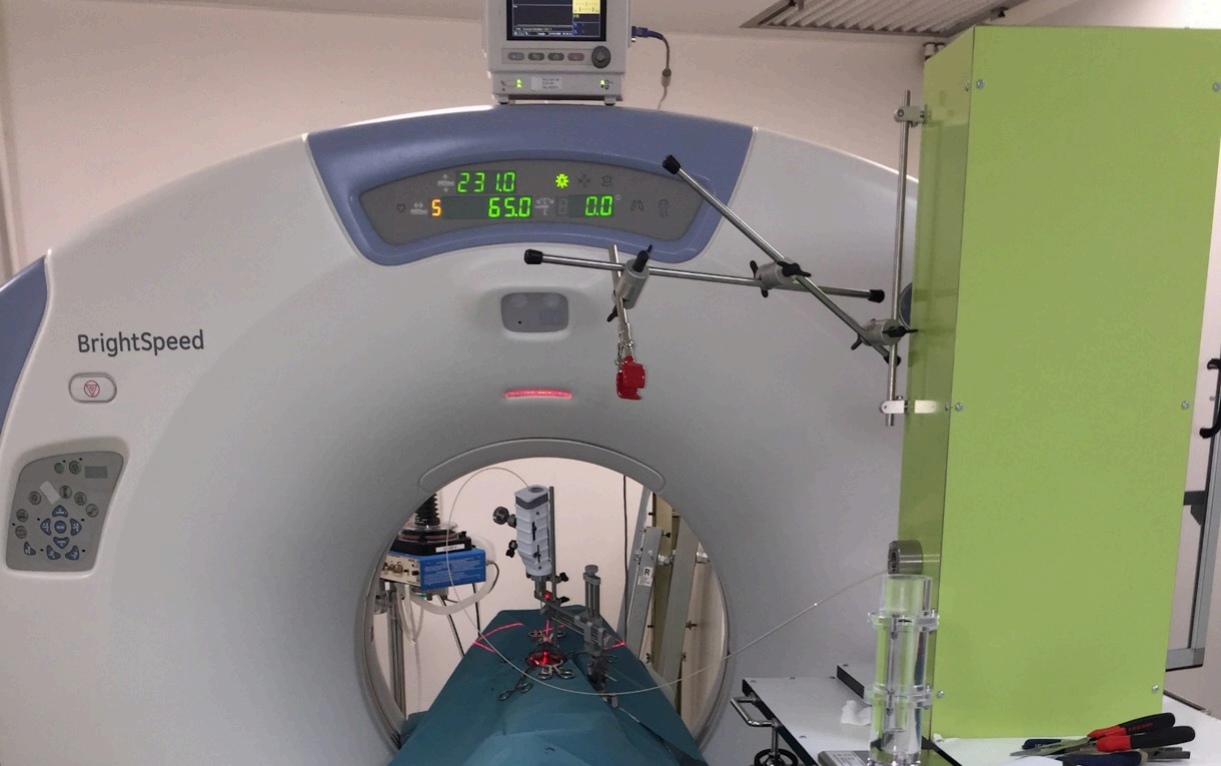
Preclinical injection system developed by EVEON. The injector is located beside the operation table in CT room. The injection needle is connected to the injector through a connection tube to transfer the Ho suspension from the injector to the needle.

The prototype injection system was developed by EVEON (Fig 1). It was designed to inject a controlled dose of radioactive Ho suspension distributed over the full volume of the tumor of large animals with unresectable brain cancers. Tumors could be between 1 and 3 cm in diameter. Larger brain tumors can be treated but, due to the mechanical specification of the injector, they would therefore have to be considered as several tumors of the size defined above. The main components of the injection system include: 1) Injector: reusable equipment used to manage the injection of a defined quantity of radioactive microparticles suspension, 2) Injector control software used to manage the injection parameters, such as injection volume, number of injections per tumor and the injection flow rate, 3) injection needle: contains a needle guide with a needle inside, used to move and access into different parts of the tumor and deliver the Ho suspension (attached to the stereotactic frame for aligning the injection needle on the planned trajectory), 4) prefilled suspension capsules: protected containers holding a volume of radioactive microparticles, 5) disposable fluidic cassette: dedicated disposable fluidic circuit controlled by the injector equipment that transfers the suspension from prefilled capsules to the injection needle. This cassette is composed of three main elements: (a) a distributor that circulates the suspension to different parts of the fluidic circuit of the injector, (b) a plunger that drives the suspension outside the capsules, (c) a connection tube that transfers the suspension from the distributor located in the injector to the injection needle. The injection technique consists of using the stereotactic frame to manually deploy the injection needle at a given position in the tumor. This trajectory is established by a cartography based on a 3D scanner. The Ho suspension is then automatically injected by the software. The injection command is executed *via* the injector control software or a surgeon-activated footswitch. This command sends an instantaneous signal, which actuates the injector pump and valves allowing the suspension to be sent to the tip of the needle and thus injected with predefined unit volume and flowrate. This operation was then repeated for all injections, without ever removing the injection needle from the tumor. In this way, there was only one penetration of needle guide into the tumor and several injections were done when the needle was out of the needle guide and inside the tumor.

### Treatment planning

As with all forms of modern RT, treatment planning (TP) is required to sufficiently irradiate the tumoral zone without over-irradiating surrounding healthy tissue. The main objectives of the TP were the tumor coverage by delivering at least 95% of the target absorbed dose, 100 Gy, to at least 95% of the tumoral volume. This coverage is ensured by the injection trajectory, the injection volume and the number of injections while minimizing this number as much as possible. The number of injections therefore depends on the shape and size of the tumors. In tumor coverage, what would matter is a beta-radiation coverage beyond a certain energy deposition threshold. More information on the bespoke microbrachytherapy TP process can be found in Brown *et al*.[53].

In the first four minipigs, the TP was first performed based on CT-scans that were acquired 10 days after the implantation (D10), meaning 4 days before the injection. But in pig n° 3 and 4, TP was modified to account for changes to the tumor depth and diameter, based on the pre-operative CT-scan acquired at day 14 post-implantation (D14), 30 minutes before the treatment. This modification was required because of a rapid increase in tumor volume and change in tumor coordinates between D10 and D14. In the fifth minipig, TP was performed directly on a CT scan that was acquired as close to the treatment as possible, 30 minutes before the ^165^Ho injection, at D14 (Table 1). Performing the CT-scan as close to the treatment as possible meant that the planning was performed with the most up-to-date information regarding the tumor’s size, shape and position.

**Table 1:** Different injection parameters for each minipig.

Pig number	1	2	3	4	5
**Volume of unit of treatment (UoT) (**μ**l)**	5	5	5	5	5 and 9^a^
**Injection rate (**μ**l/min)**	20	20	50	50	50
**Treatment planning CT acquisition**	D10	D10	D10^b^	D10^c^	D14

^a^: In pig n° 5, treatment unit was 5 μl in left and 9 μl in right tumor.

^b, c^: Treatment planning was modified on the day of Ho injections based on pre-operative CT-scan (D14)

For the CT-acquisition, contrast product was used to easily differentiate the tumor from healthy tissue. Tumor segmentation was then performed with the open-source software, 3D Slicer. Once segmentation had been completed, TP was performed with the optimization algorithm, the non-dominated sorting genetic algorithm II [54]. The volume of each injection, known as a unit of treatment (UoT) was fixed at 5 μl, except in the right tumor of pig n° 5, which was fixed at 9 μl to decrease the number of injections and reduce the duration of surgery (Table 1).

### Immunosuppression

An immunosuppressive therapy of the animals is mandatory to reduce the risk of rejection of grafted cells during the study. According to our previous studies on development of GB in Yucatan minipig and in Landrace pig, an immunosuppressive therapy was standardized for porcine glioma model [7,51]. In this way, cyclosporine (25 mg/kg; Neoral^®^ 100 mg/ml) was administered orally, twice a day. This treatment continued each day until the minipigs were euthanized. To increase the probability of tumor development, the blood level of cyclosporine was kept above 1000 ng/ml. Blood samples were taken once a week to monitor the amount of cyclosporine in the serum.

### Cell implantation and intratumoral injection of ^165^Ho

Tumors were implanted bilaterally to increase the number of tumors per animal and, consequently, to decrease (for ethical concerns) the number of enrolled animals. The tumoral cell implantation and its surgical procedure were performed as previously described [7]. All the surgeries were performed under general anesthesia. Minipigs were pre-medicated with an intramuscular (IM) injection of azaperone (Stresnil^®^) and atropine sulfate. After 15–20 min, the anesthesia was induced by IM injection of Tiletamine + Zolazepam (Zoletil^®^100). An isotonic solution was continuously administered through a catheter. Under orotracheal intubation, the sedation was maintained by inhalation of isoflurane 2% in Oxygen. The animals were given the analgesic: morphine hydrochloride (Aguettant^®^) and antibiotic treatments: Amoxicillin + Clavulanic acid (Augmentin^®^) before starting the surgery. During the operation, the functionality of the cardio-respiratory system was monitored. In the case of showing any sign of pain, morphine was administered throughout the intervention. After the surgery, the animal was transferred to the recovery room for post-operative monitoring. A pain reliever was supplied via a Fentanyl patch 25 μg/h. Prophylactic antibiotic (Kesium^®^) was administered until 6 days after the surgery. Intratumoral injections of ^165^Ho were performed on five minipigs. The injections took place fourteen days after the tumor cells implantation (D14) in which the tumor size was between 1.5 and 3 cm. In the operating room, the preclinical injection system was placed near to the animal and connected to the preclinical software. A few minutes before starting the injections, the Ho suspension was vortexed for one minute to ensure homogeneity. Then, a dedicated capsule was filled with the prepared suspension. It was placed in a specific capsule support and mounted into the injection system. The injection needle was installed in the stereotactic frame before penetrating in the brain and accessing the tumor. To access the holes that had been created for tumoral cell implantation, the incision was done exactly on the sutured line. The fatty tissue, which was placed in the holes to prevent the formation of scar tissue, was removed. According to the tumor position as determined by the pre-operative CT images (described in the following section), the needle guide was inserted into the brain. While the needle guide was inside the tumor, several UoT were injected in different sites using the coordinates given by treatment planner. The number of injections varied between tumors.

During the development of this procedure, injection parameters were optimized. For this reason, parameters were gradually changed throughout the experiment, as this study is conducted into the optimal injection configuration for microbrachytherapy. This allowed for the perfection of intratumoral administration methodologies.

In the first two minipigs, the injection rate was 20μl/min. This was then increased to 50μl/min in the remaining three minipigs to reduce operation duration (Table 1). For the last minipig (n° 5), the injection timing was modified in the injector control software by adding 5 seconds idle time after the injection command execution. This idle time is the delay between the actuation of the injector pump/valves and the moment when the Ho suspension starts to flow from the needle tip. This additional timing step was found to improve the function of injector in terms of completing the injection before withdrawing the needle. This idle time was automatically considered by the software, before each injection.

### CT-scan imaging

An anatomical CT-Scanner (GE BrightSpeed 16) was used at Voxcan (Marcy l’Etoile, France) to perform the CT acquisitions. Images were acquired under a standard protocol with a tube voltage of 140kV and a tube current of 150 mA. Voxel sizes for imaging were 0.391 mm, with a slice thickness of 0.625 mm, describing a field of view of 25 cm diameter centered on the pig’s head. The minipigs were under general anesthesia and positioned at ventral decubitus during all CT acquisitions.

On the day of cell implantation (D0), a pre-operative CT was performed just before starting the surgery, to evaluate the skull thickness and to assess the risk of any congenital problems in the brain. After the cell implantation, tumor growth was assessed with imaging on days 7, 10 and 14. To evaluate the tumor volume, a manual segmentation was performed around the tumor using version 9.2 of the Avizo software (Thermo Fisher). The segmentation was performed only on few slides and an interpolation procedure was then performed in order to obtain a Region Of Interest (ROI) around all the tumor. Using this ROI, the tumor volume was calculated in mm^3^, again with Avizo v 9.2. The day of ^165^Ho injection (D14), pre- and post-operative CT acquisitions were performed. Pre-operative CT was done to obtain the coordinates of the tumor which are necessary for preparing the TP and finding the location of injections inside the tumor. Post-operative CT was acquired to observe the distribution of injected Ho inside the tumor. The injected ^165^Ho distribution was visible through CT-scan imaging thanks to the high relative density of Ho compared to normal tissue and bone. Ho CT-scan calibration curve were determined prior to the study.

In two minipigs (n° 1 and 2) that were not euthanized directly after the post-operative CT (as described in the following section), the evolution of the injected Ho was studied by performing CT-scans at days 1 and 3 post-injection. In these minipigs, a manual segmentation of injected microparticles images was performed after each post-operative CT-acquisition. In this fashion, the movement and distribution of ^165^Ho could be studied over time.

### Euthanasia

Three minipigs (n° 3, 4, 5) were euthanized on the day of Ho injection (D14) and two (n° 1, 2) were sacrificed 3 days later at D17, after the last CT imaging to observe the distribution of Ho on macroscopic and histological samples over time. 3 days was chosen because the half-life of ^166^Ho is 26.8 hours so after this period the ^166^Ho will have released most of its radioactive energy. After about 3 half-lives, it remains only one eight of the original Ho. At the end of radiological acquisitions, while the animal was still under anesthesia, 30 ml of pentobarbital (Dolethal®) 200 mg/ml was administered intracardially. This results in an immediate and painless death.

### Histology

Histological analysis was planned to confirm the CT-scan findings. After sacrificing the minipigs, the brain was quickly removed and the tumors were fixed in 4% formalin. Fixed samples were processed and embedded in paraffin. One 4 μm section was stained with hematoxylin and eosin and histologically examined.

## Results

### Tumor growth

**Fig 2:**
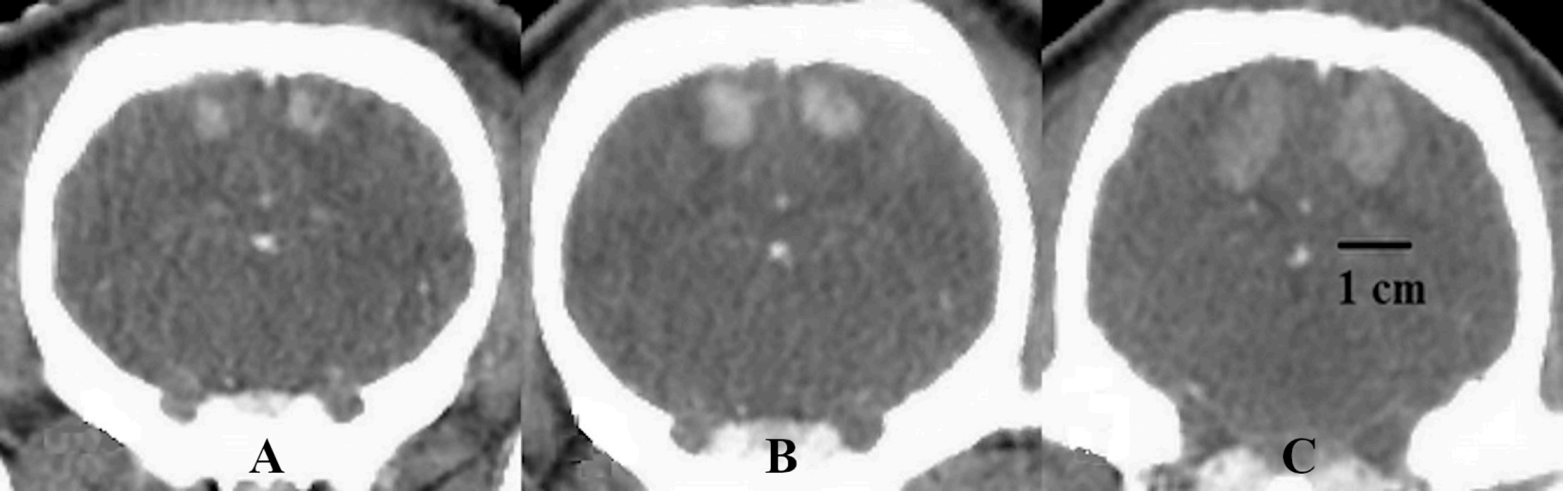
U87 GB tumor growth over time. A) Tumor size approximately 3 mm, 7 days after implantation, (B) tumor size approximately 7 mm, 10 days after implantation, (C) tumor size approximately 13 mm, 14 days after implantation, before ^165^Ho injection.

For the intratumoral injection of ^165^Ho, five minipigs were implanted with the U87 human cell line. In all animals, the subsequent CT-acquisitions after GB inoculation demonstrated tumor development in both hemispheres at the injection site in corpus striatum (Fig 2). Following the implantations, the whole-blood concentration of cyclosporine in all minipigs was above 1000 ng/ml, which prevented tumor rejection over the tumor development period. In this time, no infections or neurologic symptoms were observed. The tumor diameter at the day of Ho injection (D14) was between 1 and 1.5 cm.

### Intratumoral stereotactic injection of ^165^Ho

**Fig 3:**
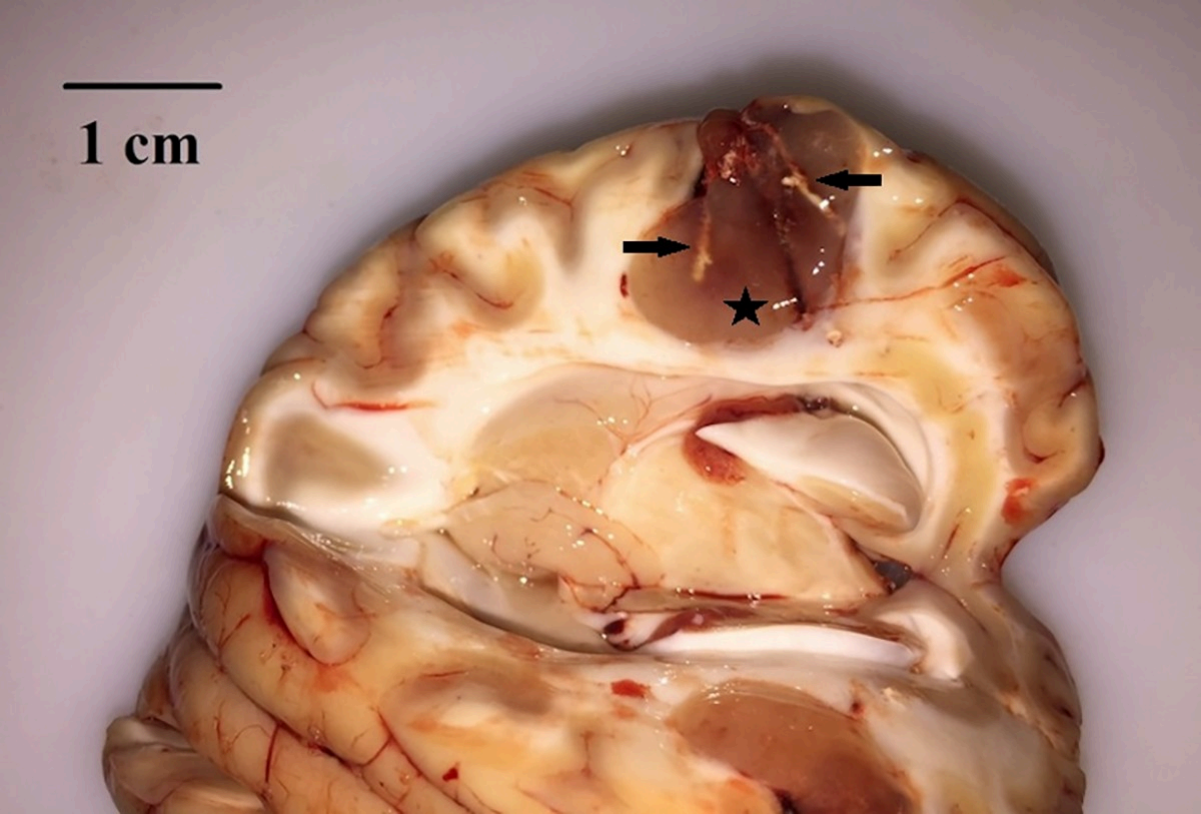
Injected ^165^Ho inside the tumor. Sagittal section of the right hemisphere. A tumor (asterisk) is present in the temporal lobe, growing from the brain surface into the gray matter and invading the white matter. White Ho suspension streaks (black arrows) are visible inside the tumor.

The intratumoral injectability of the ^165^Ho siloxane suspension was tested in preclinical studies with the minipig (Yucatan) GB model. The number of injections per tumor was computed using the tumor size and shape as input parameters (Table 2). Some parameters were modified in the injection system and TP during the test on these five animals to improve injection control and optimize the results of the intratumoral administration of the ^165^Ho. The injection rate was increased to 50μl/min in pigs 3, 4 and 5 in order to reduce operation time. After intratumoral injection of the ^165^Ho suspension, backflow was observed in the holes. This occurred after withdrawing the needle. In the first two minipigs, and especially in the left tumor of pig n° 1, the backflow volume was bigger than all the others. In pig n° 3 and 4, the backflow volume was smaller, despite increasing the injection rate to 50μl/min. In pig n°5, in which the injection timing was optimized, the quantity of Ho outside the right tumor following the 15 injections of 9μl (Table 2) (S1 Table) was nearly eliminated. After the minipigs were sacrificed and the tumors were removed, injected Ho was observed inside all the tumors (Fig 3). Macroscopically, there was no evidence of severe hemorrhage due to the needle penetration for ^165^Ho injection (Fig 3).

**Table 2:** Tumor volume (mm^3^), measured based on CT-acquisition at day 14. The number of injections per tumor was different depending on both tumor shape and size.

Pig	1		2		3		4		5	
	Left	Right	Left	Right	Left	Right	Left	Right	Left	Right
**Tumor volume (D14) mm**^**3**^	992.8	1013.9	1115.4	2019.0	966.0	878.7	758.6	797.1	871.6	2035.7
**N° of injections/ tumor**	10	9	10	15	8	8	6	5	8	15

### CT imaging and distribution of Ho microparticles

**Fig 4:**
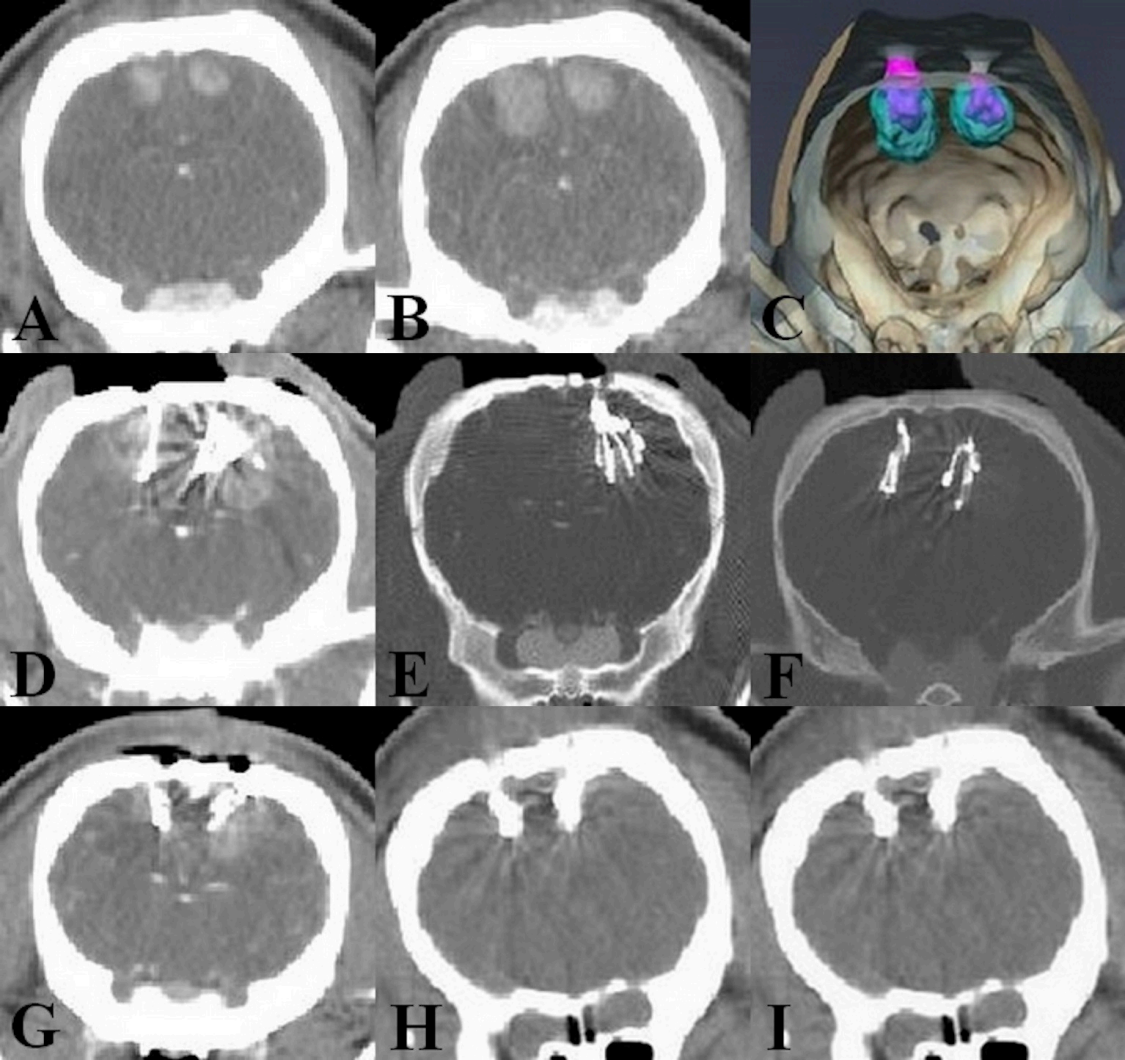
The position of Ho after the injection. (A, B) Significant tumor growth from the day of TP preparation (D10) and the day of Ho injection (D14), (C) 3D image of the injected Ho inside the tumor in pig n° 3, (D,E,F) Pig n° 5, injected Ho inside the tumor. In images E and F, the brightness is decreased to highlight volumes that receive higher concentrations of Ho, (G) Pig n° 2, injected Ho is a little in the periphery of the tumor, CT acquired at D14, (H,I) Pig n° 1, injected Ho has remained in the injection site, CT acquired 1 and 3 days after ^**165**^Ho injection.

CT images were acquired during the tumor growth period, as well as before and after Ho injections. Before injections of ^165^Ho, all the tumors displayed a slight increase in density compared to the surrounding cerebral paranchyma, with clear boundaries and a plurilobed form. There was no hemorrhage after cell implantation and a homogenous marking of the tumor. In pig n° 3 and 4, a significant tumor growth (4-5mm) was observed from the day of TP preparation (D10) and the day of Ho injection (D14) (Fig 4A and 4B), which resulted in a modification of the TP.

According to the post-operative CT-scans, the ^165^Ho suspension was successfully injected intratumorally in all five animals, especially in the last minipig. In the left tumor of pig n° 1 and both tumors in pig n° 2 (Fig 4G), the suspension was located towards the periphery of the tumor. In pig n° 3, 4 and 5, Ho was successfully injected in the desired sites (Fig 4C, 4D, 4E and 4F).

**Fig 5:**
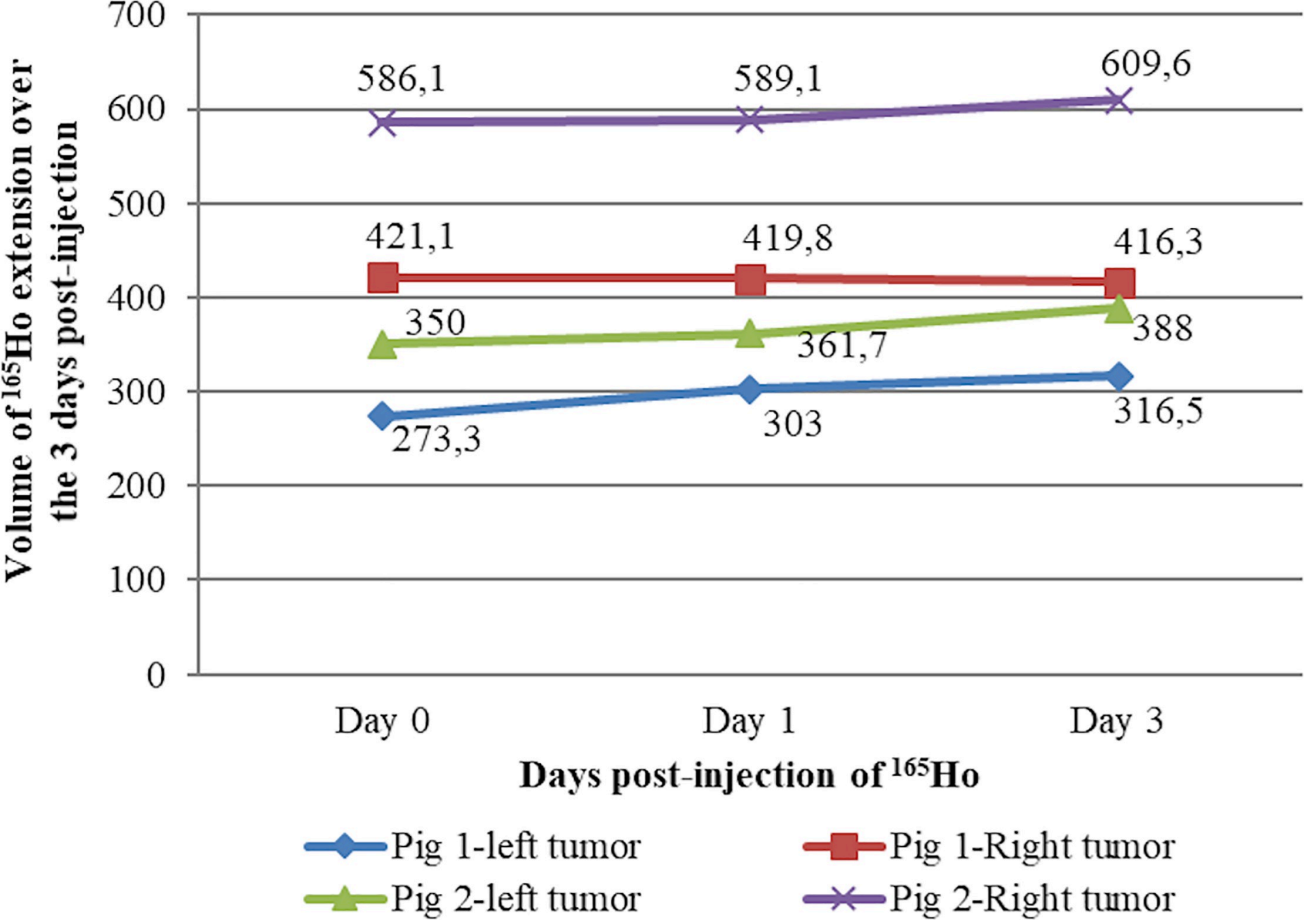
Volume (mm^3^) of distributed Ho over the 3 days post-injection (pigs n°1 and 2). Based on CT-acquisitions performed at days 0, 1 and 3 post-injection.

In the two minipigs (n° 1 and 2) with continued observation at days 1 and 3 post-injection, CT imaging showed that the Ho suspension was localized and retained at the site of injection (Fig 4H and 4I). The corresponding volume of the Ho distribution inside the tumor over time is presented in Fig 5.

### Histology

In all the examined samples, the tumor was well delimited, not encapsulated and highly cellular. It was composed of bundles of polygonal to spindle shaped cells in a scant fibrovascular stroma. Cells were moderate to large in size, with no evident cellular borders. Nucleo-cytoplasmic ratio was moderate; the cytoplasm was eosinophilic and the nucleus was round to irregular, centrally located, with a motted chromatin and 1 to 2 nucleoli. Anisocytosis and anisokaryosis were moderate to severe; some multinucleated cells and megalocytes were visible. Mitoses were up to 10 per high power field (400X), corresponding to a 2.37 mm^2^ surface. Small foci of necrosis were present. A moderate number of lymphocytes and plasma cells were present around the tumor. Pigs n° 3, 4 and 5 exhibited a moderate amount of intravascular and perivascular inflammatory cells (mainly lymphocytes, less plasma cells) within the tumor; inflammatory cells were also present around it. Refrangent, lightly brown-gold, polygonal crystals consistent with Ho, were detected in all pigs. In pig n° 1 and 2 they were present on the superficial part of the tumor, while in pigs n° 3, 4 and 5, they were located in intra-tumoral cavities.

**Fig 6:**
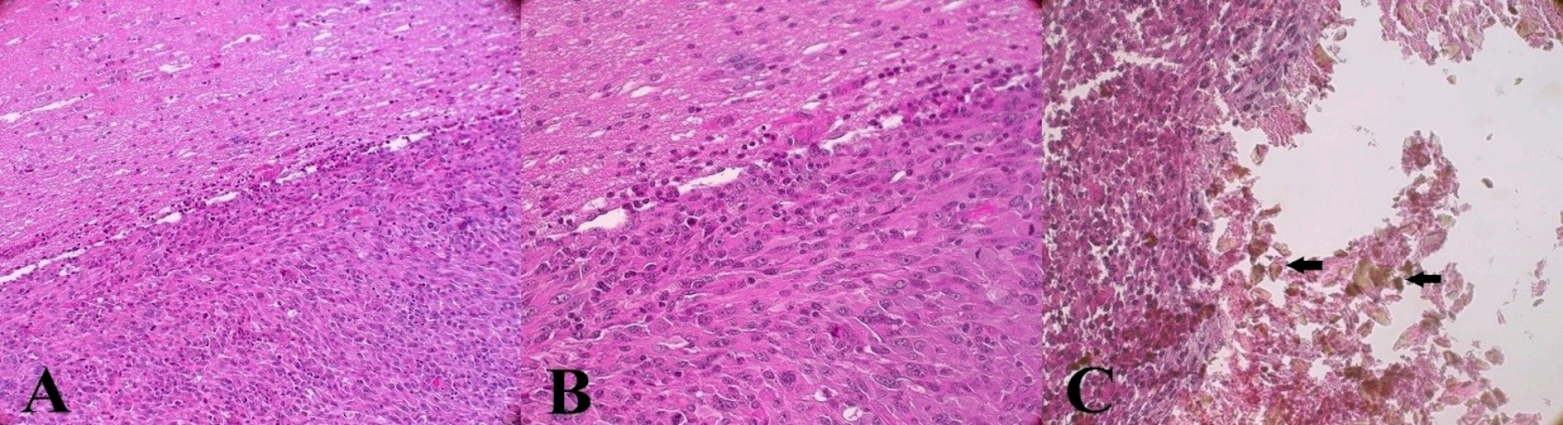
Microscopic findings of U87 tumor and injected ^165^Ho. (A) The tumor is well delimited from the adjacent parenchyma, (B) it is highly cellular, and composed of polygonal to spindle cells. (C) Photo is taken without the condenser, to better show Ho crystals. Ho is visible within the tumor as lightly brown-gold, polygonal crystals (array).

Histologically, Ho crystals were present in cavities inside the tumor (Fig 6). Mild micro-hemorrhage was occasionally observed in some samples.

## Discussion

In this experiment, the feasibility of intratumoral injection of ^165^Ho suspension was tested in Yucatan GB model.

The main pitfall of the intratumoral delivery route of anticancer agents is leakage of the injected material [55,56]. Rodent brains are lissencephalic, meaning that their brains do not contain sulci causing improvement in the drug delivery in animal experiments [57,58]. This explains why many successful treatment methods applied in rodent models fail in the human clinical phase [59]. For this reason, the Yucatan minipig was chosen as a GB model, because of the similarity with humans. Also, the minipig’s brain size makes it an appropriate model for the development and improvement of new therapeutic devices that can be designed directly at the human scale [7,51]. Following the histology of implanted tumors (Fig 6) as described in the previous study [7], tumors presented the same features as described by Jacobs *et al*. with the U87 rodent model [60]. Tumors were not capsulated but a clear demarcation with the normal parenchyma was present. The tumor presented small to moderate areas of hypoxic necrosis, lined by cells in palisade, as described by Candolfi *et al*.[61]. As with the rodent model, no glomeruloid vascular proliferation was observed [60,61]. Blood vessels were present, but they were neither hyperplastic, nor were there as many as reported by Candolfi *et al*. [61].

As described, GB remains an incurable disease in spite of all efforts made to improve current therapeutic methods [49,62]. BT is used increasingly in treatment of cerebral glioma, because it decreases side effects of other radiotherapy modalities due to irradiation of normal tissue around the tumor [49,63,64]. But in BT, a complicated surgery is needed to insert and fix the radioactive source in cerebral tumors. This can cause some secondary effects and even aggravate the neurological symptoms of the patients [33,65]. A way to overcome this issue could be the microbrachythepray method which consists of injecting a microscopic radioactive source, formulated as a suspension, directly into the tumor. In this context, the main issue is that liquid radioactive sources will potentially gelatinize and dwell in the injection area after a certain period.

In our study, ^165^Ho siloxane was used to test the feasibility of the intratumoral injection procedure and rectify any potential problems that might occur before advancing to future studies using the radioactive, therapeutic version, ^166^Ho. This source is an attractive therapeutic radionuclide due to its reasonable half-life (26.8 h), low-energy gamma ray, which is useful for imaging, and high-energy beta suitable for treatment. The advantage is that the β (-) particles have a limited penetration range in the tissue. This results in toxic irradiation of the tumor and minimal irradiation of the healthy tissue [48]. The use of Ho on cancer cells has been demonstrated in several studies. Lee *et al*. showed the radionecrosis of hepatoma, transplanted subcutaneously in the nude mouse, after an intratumoral injection of ^166^Ho [66]. Bult *et al*. have concluded that intratumoral injections of ^166^Ho Acetylacetonate Microspheres in the orthotopic mouse model of renal cancer stopped tumor growth [67]. In addition, powerful multimodal imaging opportunities such as MRI and SPECT are possible with Ho [44,45,67]. Huh *et al*. have reported that ^166^Ho can be used for the treatment of malignant glioma when injected at the appropriate dose. This suggestion was based on the results of their study on the therapeutic effects of the ^166^Ho chitosan complex on the malignant cerebral tumor model of rats [49].

In ideal conditions, the injected Ho suspension should be retained for an extended period of time to avoid its diffusion outside the tumor and also to minimize the reflux after injection [53]. In our case, Ho suspension was successfully delivered inside the tumor. As can be seen in Fig 4H and 4I, from the CT-scans of the two first minipigs 1- and 3-days post-injection, it was demonstrated that ^165^Ho siloxane is well circumscribed inside the tumor. Also, in Fig 5 the post-injection volume of ^165^Ho extension confirm that in each tumor, there was little change in the Ho distribution volume over the 3 days (S1 Fig). This is a very important point for the prevention of damage to the normal brain tissue. Suzuki *et al*. have reported that after intrahepatic administration of ^166^Ho in rats, most of the administered radioactivity was retained at the administration site for over 72 hours [68]. In Fig 5, the variation in distribution of ^165^Ho in left and right tumor relates to the difference in number of injections per tumor according to the TP (Table 2) (S1 Table). This explains the difference in initial injected volumes for pig n° 2 (350 and 586.1 mm^3^ for the left and right hemispheres, respectively).

In pig n° 1, a similar number of injections was used in the left and right tumor (Table 2), yet Fig 5 shows that the initial volume of ^165^Ho in the left and right hemispheres was 273.3 and 421.1 mm^3^, respectively. We attribute this difference to the presence of a significant reflux in the left tumor.

As shown in CT-imaging (Fig 4G) and histology of pigs n° 1 and 2, the ^165^Ho suspension was injected superficially, in the periphery of the tumor. The decreased accuracy in these 2 minipigs could be due to the tumor growth and movement during 4-day interval between preparing TP (based on the CT-scan at D10) and the Ho injection (D14). Given that ^166^Ho deposes 90% of the energy within the range of 2.1 mm and the majority of the remaining 10% in range of 2.1–8.6 mm [48–50,68], the injections in periphery of the tumor would not have been able to irradiate the totality of the targeted volume, reducing the efficacy of this treatment method.

In pig n° 3 and 4, the results were considerably better because the TPs were modified based on the pre-operative CT-scans (D14). For the pig n° 5, injected using TP that was produced using CT images taken as late as possible, the most successful positioning and distribution of Ho inside the tumor was observed. These results highlight the importance of real-time TP for this treatment method. In this case, anatomical accuracy would be improved, increasing protection of the healthy tissue and better targeting and covering of the tumoral tissue.

Furthermore, during the tests on the five minipigs, the main issue encountered was the backflow of Ho after withdrawing the needle, which sometimes even reached the holes that were drilled in the minipigs’ skulls to implant the tumors. In the treatment phase using ^166^Ho, a non-negligible presence of reflux would introduce the possibility of radioactive contamination. This showed the importance of using ^165^Ho as a non-radioactive isotope at this step.

Also, through the presence of reflux, there would be a risk that sufficient irradiation of the tumor would not be achieved if some of the radioactive products were no longer located within the tumor. Using the real-time TP had a relative effect on diminution of ^165^Ho backflow as it was observed in minipig n° 3 and 4 after modifying the TP just before the injections and in minipig n° 5 which was injected applying TP based on CT images that were acquired 30 minutes before the ^165^Ho injection. In this condition, suspension is injected correctly in depth of tumor causing reduction in risk of backflow. Also, it was found that the rest time (5 seconds idle time) of the injector before injecting each UoT had an important effect on reflux volume. This means that by adding the idle time needed for the injector before each injection, the volume of refluxed suspension can be significantly reduced. In this condition, the UoT is injected completely before withdrawing the needle and consequently well circumscribed in the injection point. With this technique, reflux in the last minipig (n° 5) was eliminated, despite increasing the flow rate to decrease the operation duration.

In addition, as can be seen in Fig 6, no evidence of severe hemorrhage was observed on the histological slides of the removed tumors. This means that the injection needle is compatible for intratumoral injection of ^165^Ho siloxane with as low risk as possible in brain damage.

In terms of fitting into the current standard care of GB, at this time this microbrachytherapy treatment is foreseen in cases of unresectable tumors and in particular for recurrent GB. In our study, the methodology has not been considered in combination with Temozolomide or after maximum safe resection of the tumor.

Further research into the combination of this method with other GB therapeutic techniques is needed.

In this study, some limitations must be taken into consideration. Firstly, it was the first time that our injection system was used for the intratumoral injection of Ho during a surgery in a large animal GB model. Throughout this study, it was inevitable that some injection parameters would change in order to optimize the injection procedure and reduce as much as possible the risks linked to radioactive^166^Ho use. In this condition, the repeated use of optimized parameters would have been extremely useful to show the consistency of the results, but we had ethical concerns in increasing the number of animals at this step of the project. Secondly, we were also limited by the number of pigs needed to evaluate the volume of ^165^Ho extension over a longer period of time and collect histological samples at many different timepoints.

## Conclusion

Through this proof of concept study on the feasibility of stereotactic multi-injection of ^165^Ho siloxane inside the tumor, it was demonstrated that ^165^Ho suspensions can be injected successfully inside the tumor with minimal or no Ho reflux after the injections. This injection technique, as well as the ^165^Ho siloxane suspension, needs to be evaluated using radioactive ^166^Ho. Before that, it was important to use ^165^Ho to minimize the risk of radioactive contamination during the ^166^Ho injections inside the tumors. The final step will be the evaluation of treatment efficacy using radioactive ^166^Ho, as microbrachytherapy, in Yucatan GB model.

## Supporting information

S1 Table Total injected volume (μl) in each tumor at day 14. The total injected volume was calculated based on volume of UoT and number of injections per tumor, depending on tumor shape and size.
(DOCX)

S1 Fig Change in Ho distribution volume over the 3 days. (TIFF)
