# Supplementary material for: Feasibility of intratumoral 165Holmium siloxane delivery to induced U87 glioblastoma in a large animal model, the Yucatan minipig
Source: PLoS One. 2020 Jun 18;15(6):e0234772. doi: 10.1371/journal.pone.0234772 (PMC7302492; doi:10.1371/journal.pone.0234772)
Supplement: S1 Table — The total injected volume was calculated based on volume of UoT and number of injections per tumor, depending on tumor shape and size. (DOCX) [file pone.0234772.s001.docx]

S1 Table: Total injected volume (µl) in each tumor at day 14. The total injected volume was calculated based on volume of UoT and number of injections per tumor, depending on tumor shape and size.

| **Pig** | **1** | | **2** | | **3** | | **4** | | **5** | |
| --- | --- | --- | --- | --- | --- | --- | --- | --- | --- | --- |
|  | Left tumor | Right tumor | Left tumor | Right tumor | Left tumor | Right tumor | Left tumor | Right tumor | Left tumor | Right tumor |
| **Tumor volume (D14) mm^3^** | 992.8 | 1013.9 | 1115.4 | 2019.0 | 966.0 | 878.7 | 758.6 | 797.1 | 871.6 | 2035.7 |
| **N° of injections/ tumor** | 10 | 9 | 10 | 15 | 8 | 8 | 6 | 5 | 8 | 15 |
| **Volume of unit of treatment (UoT) (µl)** | 5 µl | 5 µl | 5 µl | 5 µl | 5 µl | 5 µl | 5 µl | 5 µl | 5 µl | 9 µl |
| **Total injected volume (µl)/ Tumor** | 50 µl | 45 µl | 50 µl | 75 µl | 40 µl | 40 µl | 30 µl | 25 µl | 40 µl | 135 µl |
