# Supplementary figures and images for: Feasibility of intratumoral 165Holmium siloxane delivery to induced U87 glioblastoma in a large animal model, the Yucatan minipig
Source: PLoS One. 2020 Jun 18;15(6):e0234772. doi: 10.1371/journal.pone.0234772 (PMC7302492; doi:10.1371/journal.pone.0234772)

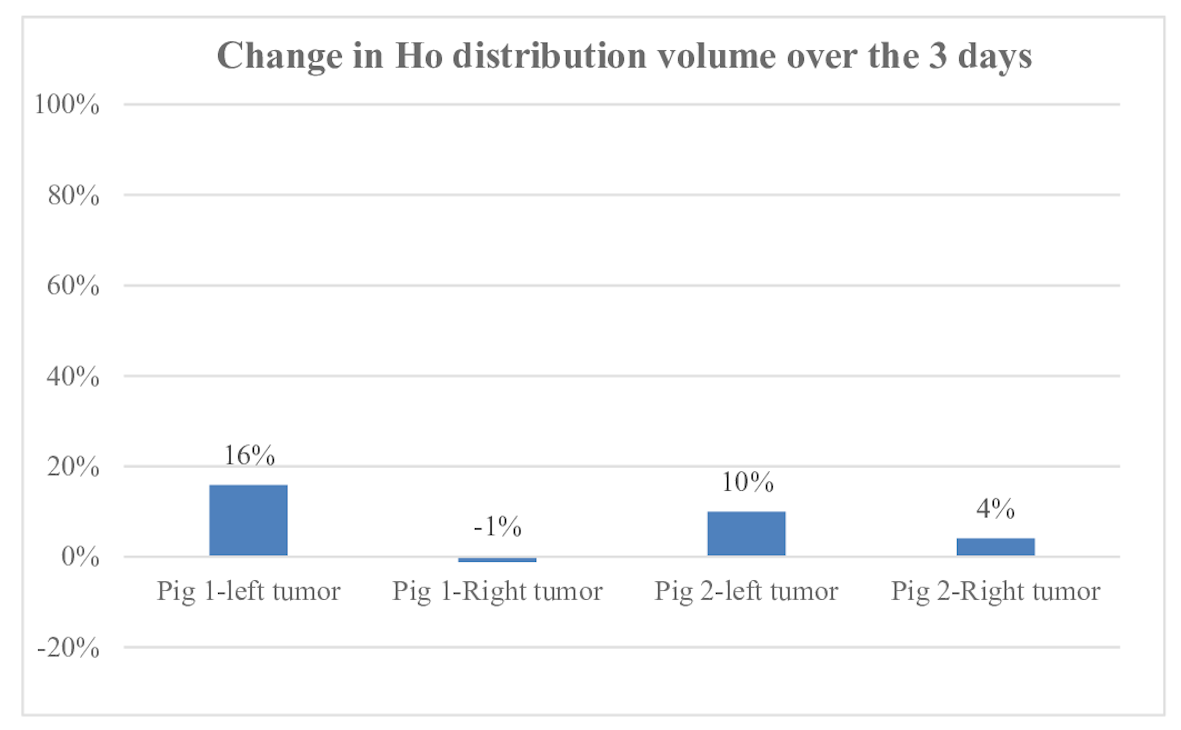

Supplement: S1 Fig — (TIFF) [file pone.0234772.s002.tiff]
